# Protocol for the systematic review of the prevention, treatment and public health management of impetigo, scabies and fungal skin infections in resource-limited settings

**DOI:** 10.1186/s13643-016-0335-0

**Published:** 2016-09-23

**Authors:** Philippa May, Asha Bowen, Steven Tong, Andrew Steer, Sam Prince, Ross Andrews, Bart Currie, Jonathan Carapetis

**Affiliations:** 1 Wesfarmers Centre for Vaccines and Infectious Diseases, Telethon Kids Institute, University of Western Australia, PO Box 855, West Perth, WA 6872 Australia; 2 Telethon Kids Institute, University of Western Australia, West Perth, Australia; 3 Menzies School of Health Research, Charles Darwin University, Casuarina, Australia; 4 Murdoch Children’s Research Institute, University of Melbourne, Parkville, Australia; 5 One Disease, Tiwi, Australia

**Keywords:** Impetigo, Scabies, Tinea, Therapeutics, Public health, Endemic diseases

## Abstract

**Background:**

Impetigo, scabies, and fungal skin infections disproportionately affect populations in resource-limited settings. Evidence for standard treatment of skin infections predominantly stem from hospital-based studies in high-income countries. The evidence for treatment in resource-limited settings is less clear, as studies in these populations may lack randomisation and control groups for cultural, ethical or economic reasons. Likewise, a synthesis of the evidence for public health control within endemic populations is also lacking. We propose a systematic review of the evidence for the prevention, treatment and public health management of skin infections in resource-limited settings, to inform the development of guidelines for the standardised and streamlined clinical and public health management of skin infections in endemic populations.

**Methods:**

The protocol has been designed in line with the Preferred Reporting Items for Systematic Reviews and Meta-Analyses Protocols statement. All trial designs and analytical observational study designs will be eligible for inclusion. A systematic search of the peer-reviewed literature will include PubMed, Excertpa Medica and Global Health. Grey literature databases will also be systematically searched, and clinical trials registries scanned for future relevant studies. The primary outcome of interest will be the clinical cure or decrease in prevalence of impetigo, scabies, crusted scabies, tinea capitis, tinea corporis or tinea unguium. Two independent reviewers will perform eligibility assessment and data extraction using standardised electronic forms. Risk of bias assessment will be undertaken by two independent reviewers according to the Cochrane Risk of Bias tool. Data will be tabulated and narratively synthesised. We expect there will be insufficient data to conduct meta-analysis. The final body of evidence will be reported against the Grades of Recommendation, Assessment, Development and Evaluation grading system.

**Discussion:**

The evidence derived from the systematic review will be used to inform the development of guidelines for the management of skin infections in resource-limited settings. The evidence derived will be intended for use by clinicians, public health practitioners and policy makers in the treatment of skin infections and the development of skin infection control programmes. The review will identify any gaps in the current evidence to provide direction for future research.

**Systematic review registration:**

PROSPERO CRD42015029453

**Electronic supplementary material:**

The online version of this article (doi:10.1186/s13643-016-0335-0) contains supplementary material, which is available to authorized users.

## Introduction

The skin infections impetigo, scabies and tinea disproportionately affect the most vulnerable populations [1–4]. Children in low-income countries, tropical regions and disadvantaged populations within high-income countries are amongst the worst affected [5–7]. Impetigo and scabies are correlated; people with scabies are at risk of secondary bacterial infection with impetigo and areas with a high prevalence of one condition often have a high prevalence of the other [5, 8]. Tinea infections (superficial mycoses) are also common in tropical and resource-limited settings and occur more frequently in children than in adult populations [9, 10]. Tinea may also be complicated by impetiginisation [9]. Without treatment of impetigo or secondarily infected dermatoses, serious and sometimes fatal complications can occur, including sepsis, invasive infection and post-streptococcal sequelae [4, 11–16].

### Impetigo

Impetigo is characterised by golden exudate and crust, typically caused by the bacteria *Staphylococcus aureus*, the prevalent bacteria in temperate regions, or *Streptococcus pyogenes* (group A beta haemolytic *Streptococcus* (GAS)), the predominant organism in tropical contexts [6, 17]. Impetigo can occur on any part of the body where breaks in skin integrity have occurred [18–20]. Infestation with the scabies mite is a major contributing factor in resource-limited settings and tropical regions [6, 12, 21]. The global paediatric population suffering from impetigo at any one time has been estimated at >162 million; but this affliction is not uniformly distributed, with children in tropical regions and resource-limited settings carrying the greatest burden of disease [22].

The medical treatment of impetigo comprises either topical or systemic antibiotics directed at the two common pathogens [23–26]. Hand hygiene [27], topical disinfectants [24, 25] and school exclusion measures [28, 29] may be used as adjuncts for treatment and contribute to disease control. Natural therapies and traditional medicines such as tea tree oil [30] and cocky apple tree poultices or suspensions [31] are used by some populations.

Koning et al. concluded that the topical antibiotics, fusidin and mupirocin, were the most effective treatment for mild to moderate impetigo [24, 25]. However, the majority of studies included in the review were conducted in high-income countries and hospital outpatient settings [24, 25]. In contrast, in Australian Aboriginal and Torres Strait Islander communities, the recommended treatment for impetigo has been intramuscular benzathine penicillin, based on non-randomised trials conducted over 40 years ago [32, 33]. Although Koning et al. did not limit the Cochrane systematic review by population and setting [24, 25], few studies were included that involved populations in resource-limited settings, as studies conducted in these settings may have been non-randomised and uncontrolled and thus did not satisfy inclusion criteria. As such, it is questionable whether the findings, namely the first-line use of topical antibiotics, can be recommended for treatment of impetigo in resource-limited settings, particularly as impetigo is often severe in these settings [21]. With recent evidence showing the efficacy of oral trimethoprim-sulfamethoxazole [21], growing concern regarding antimicrobial resistance to topical agents [34] and rising rates of methicillin-resistant *S. aureus* (MRSA) [35], a review of the evidence base for effective treatment of impetigo in resource-limited settings is needed. In addition, primordial prevention interventions for impetigo targeting the social determinants of health, which underlie the burden of skin infections across the world [1–3, 36], require review.

### Scabies

Scabies, due to infestation by the mite *Sarcoptes scabiei* var. *hominis* [4], is a human only skin disease characterised by intense itch and cutaneous eruption [37, 38]. Scratching due to scabies interrupts the usual skin barrier [37], facilitating the entry of bacteria, causing secondary bacterial infection and its subsequent sequelae [37, 38]. Scabies mites crawl, but do not fly or jump; thus transmission occurs through skin-to-skin contact and occasionally through fomite spread [39, 40] as the mite survives for several days off the human host [40].

The estimated global prevalence of scabies in 2010 was over 100 million people [41]. A recent systematic review reported the highest point prevalence of paediatric scabies in the resource-limited settings of Panama (78 % of children under age 2 years affected), Fiji (44 % of 5- to 9-year-olds affected) and Australia, where one third of Aboriginal children in remote communities are affected [42].

Clinical treatment of scabies is with topical or oral anti-parasitic agents [38, 43]. Herbal therapies and traditional medicines, including natural pyrethrins, tea tree oil [44] and emu bush [45], may also be effective, as well as culturally acceptable in certain settings. Additional methods for the prevention of transmission of scabies include barrier precautions, environmental decontamination measures [40] and multifaceted population-level approaches in endemic communities involving community education, community involvement and treatment of cases and contacts [46].

In the original Cochrane Review in 2000, Walker and Johnstone concluded that topical permethrin was superior to all other treatments for scabies [43]. In the 2007 update, oral ivermectin was supported as an effective treatment, although was not licensed for use in the treatment of scabies in many countries, and topical permethrin remained superior to oral ivermectin [38]. This systematic review needs updating to take into account the recently published mass drug administration (MDA) experimental studies [16, 47].

Beyond specific clinical treatment, a Cochrane review of measures to reduce the transmission of scabies was unable to provide recommendations due to a lack of RCTs examining these [40]. A systematic review of population-level approaches to scabies prevention and control is needed but challenging, due to lack of randomisation or comparisons of programmatic approaches to scabies management in endemic communities [48]. However, effective programmes have been delivered in Australian Aboriginal communities [49, 50]. As described by Wong et al., a programme delivered in a large Aboriginal community in the Top End of the Northern Territory involving extensive community support, education, screening of school children, MDA of permethrin and environmental health services led to a significant reduction in scabies and pyoderma prevalence at 7 [50] and 15 months [49]. As scabies is endemic in areas where overcrowding, social disruption and poverty exist [51, 52], a synthesis of the best available evidence for scabies control that determines treatment effectiveness, prevention of re-infection and cross-infection within families and communities in resource-limited settings is required [37, 43, 53].

### Crusted scabies

Whilst classical scabies infestations involve 5 to 15 scabies mites [37, 39], people with crusted (or Norwegian) scabies have hyperinfestation with thousands to millions of mites and are highly infectious [37, 38]. There is a spectrum of severity, from mild cases to severe hyperkeratotic dermatosis [37]. The crusted scabies burden in remote Aboriginal communities of the Northern Territory in Australia has been estimated at 2.4 per 1000 population (Personal communication**,** S. Prince 2016). The global burden of crusted scabies is yet to be described.

Management of crusted scabies frequently involves hospitalisation of the affected individual to prevent community spread, provide frequent topical and systemic scabicides along with application of keratolytics to manage hyperkeratosis [46, 54, 55]. Environmental measures are recommended to decontaminate the environment, where the heavy infestation of scabies mites can survive for several days in the absence of the human host, including washing of clothing and bed linen [37]. In addition, household spraying or fogging with insecticides has been recommended in some clinical guidelines to prevent transmission via fomites [56, 57].

Individuals with crusted scabies are core transmitters of scabies within households or communities [37, 47]. Thus, public health interventions that identify and treat people with crusted scabies in communities where scabies is endemic are likely to reduce the burden of scabies [53, 58]. However, social stigma associated with scabies, particularly crusted scabies, is significant and must be approached with respect and sensitivity [37, 58]. Community-based identification and management of individuals with crusted scabies within the privacy of their homes has been effective in the Northern Territory of Australia using a chronic disease model of care [58]. The high transmissibility of crusted scabies from an index case [40, 47] has implications for scabies control in communities where individuals with crusted scabies reside [47, 58]; hence, a review of the evidence for effective and socially acceptable methods of crusted scabies management and control is needed.

### Tinea/ringworm

Tinea infections are classified according to the site of the human body affected [9]. Tinea corporis of the body, tinea capitis of the scalp and tinea unguium [onychomycosis] of the nail are caused by infection with dermatophytes of the genera *Epidermophyton*, *Microsporum* and *Trichophyton* [10, 59].

Between 10 and 20 % of the world’s population are affected by fungal skin infections [10]. Estimates of the disease burden have generally focussed on the prevalence of causative organisms, rather than the clinical disease entities [60, 61]. However, the World Health Organization (WHO) has estimated that 7–33 % of children assessed in clinical surveys in developing countries are affected by tinea capitis [17], and a recent systematic review reported the global prevalence of onychomycosis as 11 % [62].

Clinical treatment for tinea infection includes oral and topical antifungals and adjunctive treatment, such as selenium sulphide shampoo [7, 9, 59]. Laser therapy for tinea unguium is emerging [63–65] but its applicability in resource-limited settings remains unknown.

Tinea is highly communicable, even amongst asymptomatic carriers [9] with community-wide implications for control programmes [17]. Identification of infected contacts is important to prevent recontamination and dissemination [17]. Additional methods for tinea control include washing and disinfection of clothing, towels, combs and brushes; discouraging sharing of these items amongst household members; and concurrent treatment of family members [9, 17].

### Rationale for a systematic review applicable to resource-limited settings

People living in regions with endemic rates of scabies and impetigo [5, 66, 67] are at substantial risk of infective complications, post-streptococcal sequelae and physical disability [68, 69]. A coordinated and evidence-based approach to treatment and population management is needed [17]. All Cochrane systematic reviews for the treatment of impetigo, scabies and tinea relate to participants in high-income countries, hospital settings and people with higher socioeconomic status [10, 24, 25, 38, 40]. As such, there is no current evidence-based consensus on the management of skin infections found in the peer-reviewed literature to support clinical and public health decision making in endemic settings. We aim to develop guidelines for the prevention, treatment and community control of impetigo, scabies and fungal infections in resource-limited settings, as the predominant skin infections in these populations [1, 17], drivers of high demand for health care [17, 28, 66] and potential precursors to rare but serious illness [13, 17]. To develop a robust guideline for common skin infections in endemic populations, a peer-reviewed, systematic review of the evidence base applicable to resource-limited settings is required. Due to the overlap between the occurrence of impetigo, scabies and fungal infections [8, 9] and the need for streamlined guidance on the management of these common conditions in resource-limited settings [17], a broad systematic review encompassing multiple conditions will be conducted. The objective of the review is to assess the evidence for treatment and public health management of impetigo, scabies, crusted scabies, tinea capitis, tinea corporis and tinea unguium in endemic settings.

## Methods

This protocol is in accordance with the Preferred Reporting items for Systematic Reviews and Meta-Analyses-Protocol (PRISMA-P) statement (Additional file 1) [70]. The systematic review will be conducted using standard systematic review methodology based on the Cochrane handbook and reported in accordance with the PRISMA statement [71, 72]. The review will be overseen by a steering group, comprised of experts in the field. The review is registered with PROSPERO (International prospective register of systematic reviews) at the National Institute for Health Research and Centre for Reviews and Dissemination (CRD) at the University of York (registration number CRD42015029453).

### Eligibility criteria

#### Types of participants and setting

The review will consider studies that include participants of any age, gender or country of origin within resource-limited settings, tropical regions, remote Aboriginal and Torres Strait Islander communities and resource-limited populations in Organisation for Economic Co-operation and Development (OECD) countries or low, low-middle and middle income countries. The review will include people who have been diagnosed with impetigo, scabies, crusted scabies, tinea capitis, tinea corporis or tinea unguium.

#### Intervention

The review will examine any clinical or public health interventions that aim to reduce skin infections. Treatment interventions may include either pharmaceutical or complementary medicines, including traditional management of skin infections in relevant to the setting. Public health interventions may include communicable disease control, primary health care delivery, environmental health, health promotion, education or community-level interventions.

#### Comparator

Studies with any type of comparator will be included.

#### Outcomes and prioritisation

The primary outcomes will include cure (i.e. resolution) or improvement in the condition. A decrease in prevalence will be a primary outcome for population-based studies. Secondary outcomes to be extracted will include the clearance of organism on microbiological testing, relief of symptoms, recurrence rate, adherence to treatment or management regimen, patient acceptability, adverse effects or the proportion of contacts diagnosed with the condition within 8 weeks of diagnosis of the index case [40].

#### Study design

Any experimental study designs will be eligible for inclusion, including RCTs, quasi-experimental, before and after studies and cross over trials. The review will also include cohort and case-control analytical study designs.

### Information sources

Online databases of the peer- reviewed literature including PubMed, Excerpta Medica Database (EMBASE) and Global Health will be searched. In addition, grey literature will be accessed via the Australian Institute of Health and Welfare (AIHW), Australian Indigenous HealthInfoNet, Informit, OaIster databases and World Health Organization (WHO) website. In addition, clinical trial registries ANZ Clinical Trials Registry, ClinicalTrials.gov and WHO International Clinical Trials Registry will be searched to identify any relevant studies underway. The reference lists of all included full-text articles will be handsearched for relevant titles for inclusion.

### Search strategy

The search strategy aims to identify all relevant treatments and public health interventions applied within a resource-limited context regardless of publication status (published, unpublished, in press or in progress). The search strategy will use a combination of keywords and subject headings relating to the setting and outcomes of interest. A sample search strategy for PubMed is included (Additional file 2). Terms will be adapted according to the unique search features of the respective databases. The search will be restricted to studies published since 1960 for feasibility. Any study published in English will be considered for inclusion in the review. Non-English language articles will be excluded due to resource constraints.

### Study records

#### Data management

Titles and abstracts will be retrieved and screened using Endnote reference management software. Full-text screening and data extraction will be managed using the web-based software platform Covidence (Veritas Health Innovation, Melbourne, VIC, Australia).

#### Selection process

Two independent reviewers will screen titles, and then abstracts, for suitability using the index terms. Following this, the full text of potentially relevant reports will be retrieved where available. Full-text reports will be assessed by two independent reviewers for compliance with eligibility criteria. Reviewers will be assigned to a subset of papers after determining that they are not either an author of the paper or involved in data collection for the study. Where possible, we will attempt to correspond with primary authors via email when eligibility for inclusion is unclear. Any disagreements that arise between the reviewers regarding the studies for final inclusion will be resolved via a consensus decision of the review steering group. Members of the review steering group who have authored a paper will be excluded from any consensus decisions in relation to a paper they have authored.

#### Data collection process

Data will be extracted using standardised forms by two independent reviewers. Any disagreements will be resolved by consensus amongst reviewers or will be referred to the review steering group.

#### Data items

Data to be extracted from included studies comprises the following: author, journal, study year or where missing year of publication, funding source, study aims, setting, study design, participants, intervention type, frequency, dose, duration, co-intervention, primary outcomes, secondary outcomes and results (including effect estimates). Details of programme evaluation will be extracted if available.

#### Risk of bias assessment

An assessment of methodological quality by the two independent reviewers performing data extraction on a study will be based on The Cochrane Collaboration’s tool for assessing risk of bias [72]. For RCTs, this will comprise the following: random sequence generation; allocation concealment; blinding of participants, personnel and outcome assessors; incomplete outcome data; reporting bias and other sources of bias that may have affected the results. As recommended by Higgins and Greene, non-RCT experimental and controlled studies will also use the domains based on The Cochrane Collaboration’s tool for assessing risk of bias [72]. The “other sources of bias” domain will include an assessment for confounding for non-RCTs. For observational studies, random sequence generation and allocation concealment will not be applicable, but other domains of blinding, incomplete outcome data, selective outcome reporting and other sources of bias will be assessed, which will include an assessment of identified and other potential confounders. Risk of bias assessments will subsequently be rated as high, low or unclear in a “Risk of bias” table. Any disagreements that arise will be resolved via a consensus decision of the review steering group.

### Data synthesis

We expect there to be insufficient data to conduct meta-analyses. Quantitative findings will be presented in narrative form including additional technical tables and figures to aid in data interpretation where appropriate. Where possible, opinion-based findings will be synthesised and presented in a “Characteristics of included information” tables or text. Results for each condition will be presented in turn.

A single, comprehensive set of synthesised quantitative findings will be presented in a “Summary of Findings” table for each condition from which to develop guidelines. The quality of the evidence will be rated using the Grading of Recommendations, Assessment, Development and Evaluation (GRADE) grading system [73].

#### Meta-biases

Studies will be included only once (i.e. multiple reports of data from the same study will be screened by author names, setting, institution, type of intervention and date and duration of the study) to reduce the risk of publication bias. We will assess for evidence of selective outcome reporting when the outcomes are described in the “Methods” section but not reported in the “Results” section of the same study report.

#### Confidence in cumulative evidence

We will assess the underlying reasons for any discrepancies in results between studies, taking into account study design characteristics and risk of bias. The strength of the overall body of evidence will be reported against the Grading of Recommendations, Assessment, Development and Evaluation (GRADE) grading system [73].

## Discussion

This review will provide a synthesis of the existing literature applicable to resource-limited settings, with GRADE evidence gradings [73], to inform the development of guidelines for the management of skin infections in endemic populations and particularly Australian Aboriginal and Torres Strait Islander communities. Recommendations for skin infection prevention, treatment, public health management and programme evaluation will be the focus of the guideline. A limitation of the review is that the methodology (i.e. the inclusion of non-randomised trials and observational studies across multiple conditions) will likely preclude formulation of overall effect estimates via meta-analysis due to the expected high heterogeneity between studies. Any evidence gaps that are exposed will highlight areas for future research. A summarised version of the review will be submitted for publication. The guidelines and summary systematic review paper will be made available on the Internet for use by clinicians and public health practitioners. In addition, policymakers and health organisations may choose to use the evidence base as a resource for the development of local treatment protocols and skin health programmes.

## Additional files

Additional file 1: PRISMA-P (Preferred Reporting Items for Systematic review and Meta-Analysis Protocols) 2015 checklist: recommended items to address in a systematic review protocol. (DOC 86 kb) Additional file 2: PubMed Search Strategy. (DOCX 26 kb)

## Abbreviations

AIHW: Australian Institute of Health and Welfare
ANZCTR: Australian New Zealand Clinical Trials Registry
EMBASE: Excerpta Medica Database
GAS: Group A *Streptococcus*
GRADE: Grades of Recommendation, Assessment, Development and Evaluation
MDA: Mass drug administration
MRSA: Methicillin-resistant *Staphylococcus aureus*
OECD: Organisation for Economic Co-operation and Development
PRISMA-P: Preferred Reporting items for Systematic Reviews and Meta-Analyses-Protocol
WHO: World Health Organization
