# Supplementary material for: Protocol for the systematic review of the prevention, treatment and public health management of impetigo, scabies and fungal skin infections in resource-limited settings
Source: Syst Rev. 2016 Sep 23;5:162. doi: 10.1186/s13643-016-0335-0 (PMC5034664; doi:10.1186/s13643-016-0335-0)
Supplement: Additional file 1: — PRISMA-P (Preferred Reporting Items for Systematic review and Meta-Analysis Protocols) 2015 checklist: recommended items to address in a systematic review protocol. (DOC 86 kb) [file 13643_2016_335_MOESM1_ESM.doc]

**Additional File 1.**

**PRISMA-P (Preferred Reporting Items for Systematic review and Meta-Analysis Protocols) 2015 checklist: recommended items to address in a systematic review protocol**

| Section and topic | Item No | Checklist item | Signpost |
| --- | --- | --- | --- |
| ADMINISTRATIVE INFORMATION | | |  |
| Title: |  |  |  |
| Identification | 1a | Identify the report as a protocol of a systematic review | P1 Protocol for the systematic review of the prevention, treatment and public health management of impetigo, scabies and fungal skin infections |
| Update | 1b | If the protocol is for an update of a previous systematic review, identify as such | Not applicable |
| Registration | 2 | If registered, provide the name of the registry (such as PROSPERO) and registration number | P3 PROSPERO registration number: CRD42015029453 |
| Authors: |  |  |  |
| Contact | 3a | Provide name, institutional affiliation, e-mail address of all protocol authors; provide physical mailing address of corresponding author | P2 Dr Philippa May (Corresponding author)  Research Fellow  Wesfarmers Centre for Vaccines and Infectious Diseases  Telethon Kids Institute  University of Western Australia  PO Box 855 West Perth Western Australia 6872 Australia  Email: Philippa.may@telethonkids.org.au    Dr Asha Bowen  Telethon Kids Institute, University of Western Australia  [Asha.bowen@telethonkids.org.au](mailto:Asha.bowen@telethonkids.org.au)  A/Prof Steven Tong  Menzies School of Health Research, Charles Darwin University  [Steven.tong@menzies.edu.au](mailto:Steven.tong@menzies.edu.au)  A/Prof Andrew Steer  Murdoch Children’s Research Institute, University of Melbourne  [andrew.steer@mcri.edu.au](mailto:andrew.steer@mcri.edu.au)  Dr Sam Prince  One Disease  [sam.prince@1disease.org](mailto:sam.prince@1disease.org)  Prof Ross Andrews  Menzies School of Health Research, Charles Darwin University  Ross.andrews@menzies.edu.eu  Prof Bart Currie  Menzies School of Health Research, Charles Darwin University  Bart.currie@menzies.edu.au  Prof Jonathan Carapetis  Telethon Kids Institute, University of Western Australia  Jonathan.carapetis.telethonkids.org.au |
| Contributions | 3b | Describe contributions of protocol authors and identify the guarantor of the review | P11. PM and AB wrote the protocol. AB and JC conceived the idea. All authors critically appraised the protocol and contributed to the design and development. All authors have approved this final manuscript. JC is guarantor. |
| Amendments | 4 | If the protocol represents an amendment of a previously completed or published protocol, identify as such and list changes; otherwise, state plan for documenting important protocol amendments | Not applicable |
| Support: |  |  |  |
| Sources | 5a | Indicate sources of financial or other support for the review | P11. The authors have not received any external funding |
| Sponsor | 5b | Provide name for the review funder and/or sponsor | Not applicable |
| Role of sponsor or funder | 5c | Describe roles of funder(s), sponsor(s), and/or institution(s), if any, in developing the protocol | Not applicable |
| INTRODUCTION | | |  |
| Rationale | 6 | Describe the rationale for the review in the context of what is already known | P3-6 Introduction |
| Objectives | 7 | Provide an explicit statement of the question(s) the review will address with reference to participants, interventions, comparators, and outcomes (PICO) | P6 The objective of the review |
| METHODS | | |  |
| Eligibility criteria | 8 | Specify the study characteristics (such as PICO, study design, setting, time frame) and report characteristics (such as years considered, language, publication status) to be used as criteria for eligibility for the review | P6 Methods |
| Information sources | 9 | Describe all intended information sources (such as electronic databases, contact with study authors, trial registers or other grey literature sources) with planned dates of coverage | P8 Information sources and Search strategy |
| Search strategy | 10 | Present draft of search strategy to be used for at least one electronic database, including planned limits, such that it could be repeated | See Additional file 2 |
| Study records: |  |  |  |
| Data management | 11a | Describe the mechanism(s) that will be used to manage records and data throughout the review | P8 Data management |
| Selection process | 11b | State the process that will be used for selecting studies (such as two independent reviewers) through each phase of the review (that is, screening, eligibility and inclusion in meta-analysis) | P8 Selection process |
| Data collection process | 11c | Describe planned method of extracting data from reports (such as piloting forms, done independently, in duplicate), any processes for obtaining and confirming data from investigators | P9 Data collection process and data items |
| Data items | 12 | List and define all variables for which data will be sought (such as PICO items, funding sources), any pre-planned data assumptions and simplifications | P9 Data items |
| Outcomes and prioritization | 13 | List and define all outcomes for which data will be sought, including prioritization of main and additional outcomes, with rationale | P7 Outcomes and prioritization |
| Risk of bias in individual studies | 14 | Describe anticipated methods for assessing risk of bias of individual studies, including whether this will be done at the outcome or study level, or both; state how this information will be used in data synthesis | P9 Risk of bias assessment |
| Data synthesis | 15a | Describe criteria under which study data will be quantitatively synthesised | P12 Data synthesis |
|  | 15b | If data are appropriate for quantitative synthesis, describe planned summary measures, methods of handling data and methods of combining data from studies, including any planned exploration of consistency (such as I^2^, Kendall’s τ) | Not applicable |
|  | 15c | Describe any proposed additional analyses (such as sensitivity or subgroup analyses, meta-regression) | Not applicable |
|  | 15d | If quantitative synthesis is not appropriate, describe the type of summary planned | P9 Data synthesis |
| Meta-bias(es) | 16 | Specify any planned assessment of meta-bias(es) (such as publication bias across studies, selective reporting within studies) | P10 Meta-biases |
| Confidence in cumulative evidence | 17 | Describe how the strength of the body of evidence will be assessed (such as GRADE) | P10 Confidence in cumulative evidence |

*From: Shamseer L, Moher D, Clarke M, Ghersi D, Liberati A, Petticrew M, Shekelle P, Stewart L, PRISMA-P Group. Preferred reporting items for systematic review and meta-analysis protocols (PRISMA-P) 2015: elaboration and explanation. BMJ. 2015 Jan 2;349(jan02 1):g7647.*
