# Supplementary material for: Protocol for the systematic review of the prevention, treatment and public health management of impetigo, scabies and fungal skin infections in resource-limited settings
Source: Syst Rev. 2016 Sep 23;5:162. doi: 10.1186/s13643-016-0335-0 (PMC5034664; doi:10.1186/s13643-016-0335-0)
Supplement: Additional file 2: — PubMed Search Strategy. (DOCX 26 kb) [file 13643_2016_335_MOESM2_ESM.docx]

**Additional File 2.**

**PubMed Search Strategy**

| **Search** | **Query** |
| --- | --- |
| #1 | ((“impetigo”[MeSH Terms] OR “impetigo”[All Fields]) OR “skin sores”[All Fields] OR (“pyoderma”[MeSH Terms] OR “pyoderma”[All Fields] NOT gangrenosum))) |
| #2 | (“scabies”[MeSH Terms] OR “scabies”[All Fields]) |
| #3 | ((“crusted scabies”[All Fields] OR “Norwegian scabies”[All Fields])) |
| #4 | (“Tinea capitis”[MeSH Terms] OR Onychomycosis[MeSH Terms] OR “tinea capitis”[All Fields] OR “tinea corporis”[All Fields] OR “tinea unguium”[All Fields] OR onychomycosis[All Fields] OR dermatophyte[All Fields] OR “ringworm”[All Fields]) |
| #5 | (((((“oceanic ancestry group”[MeSH Terms] OR “minority groups”[MeSH Terms] OR “Health Services, Indigenous”[MeSH Terms] OR “American native continental ancestry group”[MeSH Terms] OR “developing countries”[MeSH Terms] OR “poverty”[MeSH Terms] OR “social class”[MeSH Terms] OR “educational status”[MeSH Terms])) OR (“developing countr*”[All Fields] OR “Developing nation*”[All Fields] OR “poverty”[All Fields] OR “low income countries”[All Fields] OR “low income nations”[All Fields] OR “middle income countries”[All Fields] OR “middle income nations”[All Fields] OR “less developed countries”[All Fields] OR “less developed nations”[All Fields] OR “third world countries”[All Fields] OR “third world nations”[All Fields] OR “socioeconomic inequality”[All Fields] OR “socioeconomic inequalities”[All Fields] OR “living standard”[All Fields] OR “living standards”[All Fields] OR “socioeconomic factors”[All Fields] OR “low resource setting*”[All Fields])) OR (Aboriginal[All Fields] OR Aboriginal’s[All Fields] OR Aboriginality[All Fields] OR Aboriginals[All Fields] OR Aborigines[All Fields] OR Indigenous[All Fields] OR Indigenousness[All Fields] OR “Inuits”[MeSH Terms] OR “Inuits”[All Fields] OR “Inuit”[All Fields] OR Inuit’s[All Fields] OR “Inuits”[MeSH Terms] OR “Inuits”[All Fields] OR Maori[All Fields] OR Maori’s[All Fields] OR Maoris[All Fields] OR “Native American*”[All Fields] OR “American Indian*”[All Fields] OR “First Nation*”[All Fields] OR “Torres Strait Island*”[All Fields] OR Metis[All Fields] OR Amerindian[All Fields] OR “native people*”[All Fields]))) Filters: Publication date from 1960/01/01; Humans |
| #6 | (“Disease management”[MeSH Terms] OR “Therapeutics”[MeSH Terms] OR “Anti-infective Agents”[MeSH Terms] OR “Anti-bacterial Agents”[MeSH Terms] OR “Antibiotic”[All Fields] OR “Antibiotic prophylaxis”[MeSH Terms] OR “Prevention and control”[SubHeading] OR “drug therapy”[Subheading] OR clinical[Title/Abstract] OR routine[Title/Abstract] OR “pharmaceutical preparations”[MeSH Terms] OR “drug therapy”[MeSH Terms] OR treatment[Title/Abstract] OR intervention[Title/Abstract] OR therapy[Title/Abstract] OR medicine[Title/Abstract] OR management[Title/Abstract]) |
| #7 | (“Disease management”[MeSH Terms] OR “therapeutics”[MeSH Terms] OR “Anti-infective Agents”[MeSH Terms] OR “Anti-parasitic Agents”[MeSH Terms] OR “drug therapy”[MeSH Subheading] OR clinical[Title/Abstract] OR routine[Title/Abstract] OR “pharmaceutical preparations”[MeSH Terms] OR “drug therapy”[MeSH Terms] OR “antiparasitic”[All Fields] OR treatment[Title/Abstract] OR intervention[Title/Abstract] OR therapy[Title/Abstract] OR medicine[Title/Abstract] OR management[Title/Abstract]) |
| #8 | (“Disease management”[MeSH Terms] OR “therapeutics”[MeSH Terms] OR “Anti-infective Agents”[MeSH Terms] OR “Antifungal Agents”[MeSH Terms] OR “drug therapy”[MeSH Subheading] OR clinical[Title/Abstract] OR routine[Title/Abstract] OR “pharmaceutical preparations”[MeSH Terms] OR “drug therapy”[MeSH Terms] OR “antifungal”[All Fields] “antimycotic”[All Fields] OR treatment[Title/Abstract] OR intervention[Title/Abstract] OR therapy[Title/Abstract] OR medicine[Title/Abstract] OR management[Title/Abstract]) |
| #9 | ((“herbal medicine”[MeSH Terms] OR “herbalism”[All Fields] OR “complementary therapies”[MeSH Terms]) OR herbal[All Fields] OR traditional[Title/Abstract] OR complementary[Title/Abstract]) OR healer[All Fields] OR ngangkari[All Fields] OR boylyada[All Fields] OR chingaruck[All Fields] OR “witch doctor”[All Fields] OR “bush medicine”[All Fields])) |
| #10 | (“Public Health Practice”[MeSH Terms] OR “Preventive Health Service”[MeSH Terms] OR “Communicable Disease Control”[MeSH Terms] OR “Disease transmission, Infectious”[MeSH Terms] OR Fomites[MeSH Terms] OR “Prevention and Control”[Subheading] OR prophylaxis[All Fields] OR contacts[All Fields] OR “exclusion from school”[All Fields] OR “exclusion from child-care”[All Fields] OR “exclusion from work”[All Fields] OR “exclusion period”[All Fields] OR “infection control”[All Fields] OR “communicable disease control”[All Fields] OR prevention[Title/Abstract]) |
| #11 | ((“Health service” OR “Health services”[MeSH Terms]) OR “Health services administration”[MeSH Terms] OR Clinic[All Fields] OR “Delivery of health care”[MeSH Terms] OR “Clinical Service”[Title/Abstract] OR “Medical Service”[Title/Abstract] OR “Aboriginal Community Controlled Health”[All Fields] OR “Aboriginal Medical Service”[All Fields] OR “Aboriginal Controlled Health Service”[All Fields] OR “Indigenous Health Service”[All Fields] OR “Ambulatory care facilities”[MeSH Terms] OR “Primary health care”[MeSH Terms]) |
| #12 | (“Swimming pool”[All Fields] OR “Water systems”[Title/Abstract] OR “Washing machine”[All Fields] OR Cleaning[Title/Abstract] OR Hygiene[Title/Abstract] OR Sanitation[Title/Abstract OR “Health hardware”[ Title/Abstract] OR Nutrition[Title/Abstract] OR Housing[Title/Abstract] OR Ecological[Title/Abstract] OR “Household-level technologies” [Title/Abstract] OR Clothing[Title/Abstract] OR Clothing[MeSH Terms] OR Environment[Title/Abstract] OR “Environment and Public Health”[MeSH Terms] OR Ecology[MeSH Terms]) |
| #13 | (“Health promotion”[All Fields] OR “Health education”[All Fields] OR “preventive education”[All Fields] OR “Wellness program”[All Fields] OR “Health campaign”[All Fields]) |
| #14 | (Education[Title/Abstract] OR (School[All Fields] OR School[Mesh Terms]) OR teaching[Title/Abstract] OR academic[Title/Abstract] OR students[Mesh Terms] OR “School health services”[Mesh Terms]) |
| #15 | (Community[Title/Abstract] OR Group[Title/Abstract] OR Local[Title/Abstract] OR Municipal[Title/Abstract] OR “Community health clinic”[ Title/Abstract]OR “Community health services”[Mesh Terms] OR “Community integration”[Mesh Terms] OR “Community medicine”[Mesh Terms] OR “Local government”[Mesh Terms]) |
| #16 | Filters activated: Publication date from 1960/01/0, Humans, English |
| #17 | #1 AND #5 AND #6 AND #16 |
| #18 | #1 AND #5 AND #9 AND #16 |
| #19 | #1 AND #5 AND #10 AND #16 |
| #20 | #1 AND #5 AND #11 AND #16 |
| #21 | #1 AND #5 AND #12 AND #16 |
| #22 | #1 AND #5 AND #13 AND #16 |
| #23 | #1 AND #5 AND #14 AND #16 |
| #24 | #1 AND #5 AND #15 AND #16 |
| #25 | #2 AND #5 AND #7 AND #16 |
| #26 | #2 AND #5 AND #9 AND #16 |
| #27 | #2 AND #5 AND #10 AND #16 |
| #28 | #2 AND #5 AND #11 AND #16 |
| #29 | #2 AND #5 AND #12 AND #16 |
| #30 | #2 AND #5 AND #13 AND #16 |
| #31 | #2 AND #5 AND #14 AND #16 |
| #32 | #2 AND #5 AND #15 AND #16 |
| #33 | #3 AND #5 AND #7 AND #16 |
| #34 | #3 AND #5 AND #9 AND #16 |
| #35 | #3 AND #5 AND #10 AND #16 |
| #36 | #3 AND #5 AND #11 AND #16 |
| #37 | #3 AND #5 AND #12 AND #16 |
| #38 | #3 AND #5 AND #13 AND #16 |
| #39 | #3 AND #5 AND #14 AND #16 |
| #40 | #3 AND #5 AND #15 AND #16 |
| #41 | #4 AND #5 AND #8 AND #16 |
| #42 | #4 AND #5 AND #9 AND #16 |
| #43 | #4 AND #5 AND #10 AND #16 |
| #44 | #4 AND #5 AND #11 AND #16 |
| #45 | #4 AND #5 AND #12 AND #16 |
| #46 | #4 AND #5 AND #13 AND #16 |
| #47 | #4 AND #5 AND #14 AND #16 |
| #48 | #4 AND #5 AND #15 AND #16 |
